# Supplementary material for: Postnatal mechanical loading drives adaptation of tissues primarily through modulation of the non-collagenous matrix
Source: eLife. 2020 Oct 16;9:e58075. doi: 10.7554/eLife.58075 (PMC7593091; doi:10.7554/eLife.58075)
Supplement: Supplementary file 1. [file elife-58075-supp1.docx]

**Supplementary File 1. Histologic variables used in the H&E scoring of the SDFT and CDET sections and the analysis method and reporting criteria adopted.** When software was adopted to acquire a parameter, the software is additionally reported. “Scored” is used to denote parameters analysed by blinded investigators.

| **Histologic variable** | **Method** | **Criteria** |
| --- | --- | --- |
| **Fascicle** |  |  |
| Cellularity | measured (HistoQuest) | 0 – 3, fewer - more |
| Nucleus shape | scored | 0 – 3, elongated to rounded |
| Organisation of collagen fibres | scored | 0 – 3, linear to non-linear |
| Crimp angle | measured (PhotoShop) | 0 – 3, smaller to larger |
| **IFM** |  |  |
| Percentage of IFM | measured (HistoQuest) | 0 – 3, lower to higher |
| IFM width | measured (HistoQuest) | 0 – 3, smaller to larger |
| Cellularity | measured (HistoQuest) | 0 – 3, fewer - more |
