# Supplementary material for: Postnatal mechanical loading drives adaptation of tissues primarily through modulation of the non-collagenous matrix
Source: eLife. 2020 Oct 16;9:e58075. doi: 10.7554/eLife.58075 (PMC7593091; doi:10.7554/eLife.58075)
Supplement: Supplementary file 2. [file elife-58075-supp2.docx]

**Supplementary File 2. Gene primer sequences used in relative mRNA expression analysis.**

| **Gene** | **Primer sequence** |
| --- | --- |
| DCN | F: CATCCAGGTTGTCTACCTTCATAACA R: CCAGGTGGGCAGAAGTCATT |
| FMOD | F: CTTGGCTCCAGACCCTGAAA  R: TGCCCCTCGCGTCAGA |
| BGN | F: TCACCTTCCAGCCCCTAGAGT  R: AGAAGCAGCCCCTCCTCAA |
| COMP | F: GGTGCGGCTGCTATGGAA  R: CCAGCTCAGGGCCCTCAT |
| COL1A1 | F: GACTGGCAACCTCAAGAAGG  R: CAATATCCAAGGGAGCCACA |
| COL1A2 | F: GCACATGCCGTGACTTGAGA  R: CATCCATAGTGCATCCTTGATTAGG |
| COL3A1 | F: ACGCAAGGCCGTGAGACTA  R: TGATCAGGACCACCAACATCA |
| TGFB1 | F: CCCTGCCCCTACATTTGGA  R: CGGGTTGTGCTGGTTGTACA |
| GAPDH | F: GCATCGTGGAGGGACTCA  R: GCCACATCTTCCCAGAGG |
| RPS20 | F: TTTGGAGAAGGTGTGTGCTGA  R: GTCTTGGTGGGCATCCGAA |
