# Supplementary material for: Postnatal mechanical loading drives adaptation of tissues primarily through modulation of the non-collagenous matrix
Source: eLife. 2020 Oct 16;9:e58075. doi: 10.7554/eLife.58075 (PMC7593091; doi:10.7554/eLife.58075)
Supplement: Supplementary file 3. [file elife-58075-supp3.docx]

**Supplementary File 3. Samples used for analysis along with statistical test used for analysis. n numbers represent biological replicates.**

| **Analysis** | **Tendon** | **Foetus** | **0 days** | **0-1 month** | **3-6 months** | **1-2 years** | **Statistical analysis** |
| --- | --- | --- | --- | --- | --- | --- | --- |
| **Biomechanics** | SDFT fascicle | n=4 | n=4 | n=3 | n=4 | n=4 | Two-way ANOVA. Outliers < or > mean ± 2xSD were removed. The average of 4-7 technical replicates was used (depending on sample availability). |
|  | SDFT IFM | n=4 | n=4 | n=3 | n=4 | n=4 |  |
|  | CDET fascicle | n=4 | n=4 | n=3 | n=4 | n=4 |  |
|  | SDFT IFM | n=3 | n=4 | n=3 | n=4 | n=4 |  |
| **Histology and Immunohistochemistry** | SDFT | n=3 | n=3 | n=3 (0-1 year) | n=4 (hist.), 3 (IHC) | | Kruskal-Wallis, Rank Sum Test |
|  | CDET | n=3 | n=3 | n=3 (0-1 year) | n=4 (hist.), 3 (IHC) | |  |
| **Elastin quantification** | SDFT | n=3 | n=3 | n=3 (0-1 year) | n=3 | | Two-way ANOVA. The average of 2 technical replicates was used. |
|  | CDET | n=3 | n=3 | n=3 (0-1 year) | n=3 | |  |
| **Proteomics** | SDFT fascicle | n=3 | n=4 | n=3 | n=4 | n=4 | Differentially abundant proteins – PEAKS Q  Differential total neopeptide abundance – One-way ANOVA, Benjamini-Hochberg correction |
|  | SDFT IFM | n=4 | n=4 | n=3 | n=4 | n=4 |  |
| **Relative mRNA expression** | SDFT | - | n=4 | n=3 | n=4 | n=4 | Two-way ANOVA |
|  | CDET | - | n=4 | n=3 | n=4 | n=4 |  |
