# Supplementary material for: Postnatal mechanical loading drives adaptation of tissues primarily through modulation of the non-collagenous matrix
Source: eLife. 2020 Oct 16;9:e58075. doi: 10.7554/eLife.58075 (PMC7593091; doi:10.7554/eLife.58075)
Supplement: Supplementary file 4. [file elife-58075-supp4.docx]

**Supplementary File 4. Collagens and proteoglycans identified in SDFT IFM and fascicle** (1% FDR, protein -10lgP >20, and ≥2 unique peptides).

| **Collagens** | **IFM** | **Fascicle** | **Proteoglycans** | **IFM** | **Fascicle** |
| --- | --- | --- | --- | --- | --- |
| COL1A1 | + | + | ACAN | + | + |
| COL1A2 | + | + | ASPN | + | + |
| COL2A1 | + | + | BGN | + | + |
| COL3A1 | + | + | CHAD | - | + |
| COL4A1 | + | - | DCN | + | + |
| COL4A2 | + | - | FMOD | + | + |
| COL4A3 | - | + | HAPLN1 | + | + |
| COL4A6 | + | - | HSPG2 | + | + |
| COL5A1 | + | + | KERA | + | + |
| COL5A2 | + | + | LUM | + | + |
| COL5A3 | + | - | OGN | + | + |
| COL6A1 | + | + | PRELP | + | + |
| COL6A2 | + | + | VCAN | + | - |
| COL6A3 | + | + |  |  |  |
| COL8A1 | + | - |  |  |  |
| COL11A1 | + | + |  |  |  |
| COL12A1 | + | + |  |  |  |
| COL14A1 | + | + |  |  |  |
| COL15A1 | + | + |  |  |  |
| COL17A1 | - | + |  |  |  |
| COL18A1 | + |  |  |  |  |
| COL21A1 | + | + |  |  |  |
| COL28A1 | + |  |  |  |  |
