# Supplementary material for: Postnatal mechanical loading drives adaptation of tissues primarily through modulation of the non-collagenous matrix
Source: eLife. 2020 Oct 16;9:e58075. doi: 10.7554/eLife.58075 (PMC7593091; doi:10.7554/eLife.58075)
Supplement: Supplementary file 5. [file elife-58075-supp5.docx]

**Supplementary File 5. Correlation analysis of IFM protein abundance and mechanical properties across development.** *p*<0.05 is highlighted in bold.

| **Correlation coeff/P value** | **Hysteresis (%)** | **Stress relaxation (%)** | **Extension at failure (mm)** | **Force at failure (N)** | **Maximum stiffness (N/mm)** | **Extension at yield point (mm)** | **Force at yield point (N)** |
| --- | --- | --- | --- | --- | --- | --- | --- |
|  |  |  |  |  |  |  |  |
| COL21A1 | **-0.98** | -0.60 | -0.76 | 0.63 | 0.89 | 0.85 | **0.98** |
|  | **0.02** | 0.40 | 0.24 | 0.37 | 0.12 | 0.15 | **0.02** |
| COL3A1 | **-0.99** | -0.67 | -0.73 | 0.67 | 0.90 | 0.80 | **0.99** |
|  | **0.02** | 0.33 | 0.27 | 0.33 | 0.10 | 0.20 | **0.01** |
| COL5A1 | **-0.96** | -0.52 | -0.78 | 0.59 | 0.86 | 0.89 | **0.96** |
|  | **0.04** | 0.48 | 0.22 | 0.41 | 0.14 | 0.12 | **0.04** |
| DCN | **-0.96** | -0.60 | -0.81 | 0.57 | 0.85 | 0.86 | **0.98** |
|  | **0.04** | 0.40 | 0.19 | 0.43 | 0.15 | 0.14 | **0.02** |
| LUM | **-0.96** | -0.67 | -0.80 | 0.58 | 0.85 | 0.82 | **0.99** |
|  | **0.04** | 0.33 | 0.20 | 0.42 | 0.15 | 0.18 | **0.01** |
| OGN | **-1.00** | -0.69 | -0.58 | 0.80 | **0.97** | 0.71 | **0.97** |
|  | **0.00** | 0.31 | 0.42 | 0.20 | **0.03** | 0.29 | **0.04** |
| PRELP | **-1.00** | -0.74 | -0.65 | 0.75 | 0.94 | 0.72 | **0.99** |
|  | **0.01** | 0.26 | 0.35 | 0.25 | 0.06 | 0.28 | **0.01** |
| COMP | -0.86 | -0.65 | -0.87 | 0.42 | 0.71 | 0.80 | 0.95 |
|  | 0.14 | 0.35 | 0.13 | 0.59 | 0.29 | 0.20 | 0.05 |
| DPT | **-1.00** | -0.75 | -0.62 | 0.77 | **0.96** | 0.70 | **0.99** |
|  | **0.00** | 0.25 | 0.38 | 0.23 | **0.04** | 0.30 | **0.01** |
| TGFBI | **-0.98** | -0.63 | -0.50 | 0.84 | **0.98** | 0.68 | 0.92 |
|  | **0.02** | 0.37 | 0.50 | 0.16 | **0.02** | 0.32 | 0.08 |
| FBLN5 | 0.82 | **0.98** | 0.38 | -0.77 | -0.83 | -0.32 | -0.86 |
|  | 0.18 | **0.02** | 0.63 | 0.23 | 0.17 | 0.69 | 0.14 |
| COL6A1 | -0.92 | -0.65 | -0.27 | 0.94 | **0.98** | 0.49 | 0.81 |
|  | 0.08 | 0.36 | 0.73 | 0.06 | **0.02** | 0.51 | 0.19 |
| COL6A2 | -0.93 | -0.70 | -0.29 | 0.94 | **0.99** | 0.49 | 0.84 |
|  | 0.07 | 0.30 | 0.71 | 0.06 | **0.01** | 0.51 | 0.16 |
| S100A4 | -0.65 | -0.56 | 0.20 | **0.96** | 0.82 | 0.07 | 0.49 |
|  | 0.35 | 0.44 | 0.81 | **0.04** | 0.18 | 0.93 | 0.51 |
| COL14A1 | 0.76 | 0.74 | 0.79 | -0.36 | -0.62 | -0.63 | -0.89 |
|  | 0.24 | 0.26 | 0.21 | 0.64 | 0.38 | 0.37 | 0.12 |
| ASPN | 0.70 | 0.90 | 0.55 | -0.48 | -0.63 | -0.37 | -0.82 |
|  | 0.30 | 0.10 | 0.45 | 0.52 | 0.37 | 0.63 | 0.18 |
| FMOD | 0.47 | 0.73 | 0.60 | -0.16 | -0.34 | -0.31 | -0.64 |
|  | 0.53 | 0.27 | 0.41 | 0.84 | 0.66 | 0.69 | 0.36 |
| KERA | 0.77 | 0.84 | 0.68 | -0.45 | -0.66 | -0.53 | -0.88 |
|  | 0.24 | 0.16 | 0.32 | 0.55 | 0.34 | 0.48 | 0.12 |
| Supplementary File 5 continued | | | | | | | |
| **Correlation coeff/P value** | **Hysteresis (%)** | **Stress relaxation (%)** | **Extension at failure (mm)** | **Force at failure (N)** | **Maximum stiffness (N/mm)** | **Extension at yield point (mm)** | **Force at yield point (N)** |
| COL1A2 | -0.47 | -0.16 | 0.25 | 0.77 | 0.64 | 0.11 | 0.27 |
|  | 0.53 | 0.84 | 0.75 | 0.23 | 0.36 | 0.89 | 0.73 |
| COL2A1 | -0.47 | -0.21 | 0.29 | 0.80 | 0.65 | 0.06 | 0.27 |
|  | 0.53 | 0.79 | 0.71 | 0.20 | 0.35 | 0.94 | 0.73 |
| COL4A1 | -0.59 | -0.35 | 0.19 | 0.88 | 0.76 | 0.14 | 0.41 |
|  | 0.41 | 0.65 | 0.81 | 0.12 | 0.24 | 0.86 | 0.59 |
| COL4A2 | -0.72 | -0.37 | 0.00 | 0.88 | 0.84 | 0.33 | 0.54 |
|  | 0.28 | 0.63 | 1.00 | 0.12 | 0.16 | 0.67 | 0.46 |
| COL6A3 | -0.81 | -0.52 | -0.09 | 0.93 | 0.92 | 0.37 | 0.66 |
|  | 0.19 | 0.48 | 0.91 | 0.07 | 0.08 | 0.63 | 0.34 |
| BGN | -0.16 | 0.05 | 0.53 | 0.59 | 0.37 | -0.17 | -0.06 |
|  | 0.84 | 0.95 | 0.47 | 0.41 | 0.63 | 0.83 | 0.94 |
| HSPG2 | -0.48 | -0.38 | 0.36 | 0.87 | 0.68 | -0.06 | 0.29 |
|  | 0.52 | 0.62 | 0.64 | 0.13 | 0.32 | 0.94 | 0.71 |
| COL5A2 | -0.93 | -0.48 | -0.86 | 0.49 | 0.80 | 0.92 | 0.95 |
|  | 0.07 | 0.52 | 0.14 | 0.51 | 0.20 | 0.08 | 0.05 |
| ADIPOQ | -0.15 | -0.02 | 0.58 | 0.62 | 0.38 | -0.23 | -0.06 |
|  | 0.85 | 0.98 | 0.42 | 0.38 | 0.63 | 0.77 | 0.94 |
| FBN1 | -0.50 | 0.06 | -0.02 | 0.58 | 0.59 | 0.40 | 0.31 |
|  | 0.50 | 0.94 | 0.98 | 0.42 | 0.42 | 0.60 | 0.69 |
| FN1 | -0.47 | -0.05 | 0.17 | 0.69 | 0.61 | 0.22 | 0.26 |
|  | 0.53 | 0.95 | 0.83 | 0.31 | 0.39 | 0.79 | 0.74 |
| FGB | 0.39 | 0.86 | 0.28 | -0.31 | -0.35 | -0.01 | -0.53 |
|  | 0.62 | 0.15 | 0.72 | 0.69 | 0.65 | 0.99 | 0.47 |
| FGG | 0.26 | 0.77 | 0.26 | -0.16 | -0.21 | 0.05 | -0.43 |
|  | 0.74 | 0.23 | 0.74 | 0.84 | 0.79 | 0.95 | 0.58 |
| LAMB2 | -0.21 | -0.11 | 0.56 | 0.69 | 0.44 | -0.23 | 0.00 |
|  | 0.79 | 0.89 | 0.44 | 0.31 | 0.56 | 0.77 | 1.00 |
| LAMC1 | -0.17 | 0.03 | 0.53 | 0.60 | 0.38 | -0.17 | -0.05 |
|  | 0.83 | 0.97 | 0.48 | 0.40 | 0.62 | 0.83 | 0.95 |
| NID1 | 0.15 | 0.31 | 0.69 | 0.33 | 0.06 | -0.35 | -0.36 |
|  | 0.85 | 0.70 | 0.31 | 0.68 | 0.94 | 0.65 | 0.64 |
| ANXA4 | -0.40 | -0.44 | 0.47 | 0.87 | 0.63 | -0.21 | 0.23 |
|  | 0.60 | 0.56 | 0.53 | 0.13 | 0.37 | 0.80 | 0.77 |
