# Supplementary material for: Postnatal mechanical loading drives adaptation of tissues primarily through modulation of the non-collagenous matrix
Source: eLife. 2020 Oct 16;9:e58075. doi: 10.7554/eLife.58075 (PMC7593091; doi:10.7554/eLife.58075)
Supplement: Supplementary file 6. [file elife-58075-supp6.docx]

**Supplementary File 6. Correlation analysis of TGFB1 whole tendon mRNA expression and IFM protein abundance across development.** *p*<0.05 is highlighted in bold.

| SDFT TGFB1 | **COL4A1** | **COL4A2** | **COL1A2** | **COL2A1** | **FN1** | **COL6A3** |
| --- | --- | --- | --- | --- | --- | --- |
| Correlation Coeff. | **0.99** | **0.99** | **0.98** | **0.98** | **0.97** | **0.96** |
| P value | **0.01** | **0.01** | **0.02** | **0.02** | **0.04** | **0.04** |
| SDFT TGFB1 | **HSPG2** | S100A4 | FBN1 | COL6A1 | LAMB2 | ANXA4 |
| Correlation Coeff. | **0.95** | 0.95 | 0.91 | 0.88 | 0.88 | 0.88 |
| P value | **0.05** | 0.05 | 0.09 | 0.12 | 0.12 | 0.12 |
| SDFT TGFB1 | BGN | LAMC1 | ADIPOQ | COL6A2 | TGFBI | NID1 |
| Correlation Coeff. | 0.87 | 0.87 | 0.86 | 0.84 | 0.76 | 0.67 |
| P value | 0.14 | 0.13 | 0.14 | 0.16 | 0.24 | 0.33 |
| SDFT TGFB1 | OGN | DPT | PRELP | COL5A1 | COL21A1 | COL3A1 |
| Correlation Coeff. | 0.66 | 0.58 | 0.56 | 0.52 | 0.51 | 0.50 |
| P value | 0.34 | 0.42 | 0.44 | 0.48 | 0.50 | 0.50 |
| SDFT TGFB1 | SERPINH1 | DCN | COL5A2 | LUM | FGG | FBLN5 |
| Correlation Coeff. | 0.49 | 0.42 | 0.40 | 0.38 | 0.37 | -0.36 |
| P value | 0.51 | 0.58 | 0.60 | 0.62 | 0.63 | 0.64 |
| SDFT TGFB1 | FMOD | FGB | COMP | KERA | COL14A1 | ASPN |
| Correlation Coeff. | 0.32 | 0.23 | 0.15 | -0.04 | 0.01 | -0.01 |
| P value | 0.68 | 0.77 | 0.85 | 0.96 | 0.99 | 0.99 |
